# AmbuBox: A Fast-Deployable Low-Cost Ventilator for COVID-19 Emergent Care

**DOI:** 10.1177/2472630320953801

**Published:** 2020-09-03

**Authors:** Zecong Fang, Andrew I. Li, Hongcheng Wang, Ruoyu Zhang, Xiyan Mai, Tingrui Pan

**Affiliations:** 1 Micro-Nano Innovations (MiNI) Laboratory, Department of Biomedical Engineering, University of California, Davis, CA, USA; 2 Department of Surgery, University of California Davis Medical Center (UCDMC), Sacramento, CA, USA; 3 School of Mechanical Engineering, Hangzhou Dianzi University, Hangzhou, China; 4 Department of Electrical and Computer Engineering, University of California, Davis, CA, USA

**Keywords:** ventilator, AmbuBag, bag valve mask, pneumatic, COVID-19

## Abstract

We present a low-cost clinically viable ventilator design, AmbuBox, using a controllable pneumatic enclosure and standard manual resuscitators that are readily available (AmbuBag), which can be rapidly deployed during pandemic and mass-casualty events with a minimal set of components to manufacture and assemble. The AmbuBox is designed to address the existing challenges presented in the existing low-cost ventilator designs by offering an easy-to-install and simple-to-operate apparatus while maintaining a long lifespan with high-precision flow control. As an outcome, a mass-producible prototype of the AmbuBox has been devised, characterized, and validated in a bench test setup using a lung simulator. This prototype will be further investigated through clinical testing. Given the potentially urgent need for inexpensive and rapidly deployable ventilators globally, the overall design, operational principle, and device characterization of the AmbuBox system have been described in detail with open access online. Moreover, the fabrication and assembly methods have been incorporated to enable short-term producibility by a generic local manufacturing facility. In addition, a full list of all components used in the AmbuBox has been included to reflect its low-cost nature.

## Introduction

As of June 15, 2020, the total number of cases of coronavirus disease 2019 (COVID-19) surpassed 7,900,000 with more than 430,000 fatalities worldwide.^1^ Acute respiratory distress syndrome (ARDS) has so far been the most common complication in COVID-19 patients requiring intensive care unit (ICU) admission.^2–6^ While mild cases of ARDS may respond to non-invasive ventilation (NIV) such as high-flow nasal cannula, moderate to severe cases of ARDS often require intubation and ventilator support.^7^ Current projections estimate that up to 1 million ventilators are needed in the United States to manage the COVID-19 pandemic, not including the anticipated surges expected in the coming months as quarantining restrictions are lifted. This is much higher than the number of ventilators estimated to be available in the United States, ranging from 60,000 to 160,000 units.^8^ Furthermore, many US hospitals are estimated to be functioning currently at or near their ventilator capacity prior to this pandemic.^9,10^

Mechanical ventilation, originally developed in the early twentieth century within the context of the polio pandemic, has been revolutionary and evolutionary with respect to delivering optimized respiratory care for critically ill patients.^11^ While mechanical ventilators have been significantly improved, they have also become more complicated. This is a natural consequence of “feature creep,” the addition of features into a device to improve the experience of users, such as healthcare providers and patients. Although this is considered a process that improves the overall function of ventilators, feature creep in fact increases the complexity of the machines in a nonlinear way.^12^ For every added feature, numerous additional algorithms and software adjustments need to be made to accommodate this feature, making regulatory hurdles more challenging, and contributing to the overall increasing cost and building complexity of the machines. As a result, a modern ventilator typically used in an ICU can cost between $25,000 and $50,000.^12^ When considering new medical equipment, however, evidence-based, patient-centered value is of utmost importance,^13^ and not all added premium features on modern ventilators have data-driven evidence to show added clinical benefits. Such features may be instead economically driven, especially when considering that in the United States, there seems to be a predilection in obtaining the latest medical technology just because it is available.^14^

In contrast, the need and use of ventilators in developing countries and rural settings is also a challenging issue that has and will continue to exist. Shah et al. studied trauma and critical care–related issues in Nepal, where they noted that electrical shortage was a major infrastructural deficiency that existed in all parts of the country.^15^ With respect to equipment, lack of pulse oximetry and ventilators was a problem in most hospitals, both large and small.

To address these issues, three categories of low-cost ventilators are currently under active development: sleep apnea machines converted to ventilators, conventional ventilator designs using components from nonclinical supply chains, and bag valve mask (BVM)-based (AmbuBag) ventilators.^16^

In the first category, commercial sleep apnea machines are converted into ventilators by adding an oxygen concentrator and virus filter. Both CPAP (continuous positive airway pressure) and BiPAP (bilevel positive airway pressure) machines have been studied. Researchers and clinicians from University of California, Berkeley and University of California, San Francisco have launched a project (Ventilator SOS) to promote the donation of sleep apnea machines to convert into ventilators for the COVID-19 pandemic.^17^ ResMed and GE Healthcare have received US Food and Drug Administration (FDA) Emergency Use Authorization (EUA) to use their sleep apnea machines for ventilator development in the COVID-19 pandemic, among a list of others.^18^ Sleep apnea machines are expensive, however, and can be difficult to access in resource-deprived settings, and this limits the ability of a prototype of this nature to be rapidly deployed.

In the second category, researchers are designing conventional ventilators with off-the-shelf components from nonclinical supply chains. A number of academic groups and nonmedical companies have led the efforts in this direction, including VITAL (Ventilator Intervention Technology Accessible Locally) from the Jet Propulsion Laboratory of NASA,^19^ Vent4US from Stanford University,^20^ Mechanical Ventilator Milano (MVM) from Elemaster S.p.A. Tecnologie Elettroniche,^21^ RespiraWorks from UC Berkeley,^22^ the European Organization for Nuclear Research (CERN),^23^ and so on. Among them, VITAL and MVM devices have recently received FDA EUA.^18^ This design strategy simplifies existing ventilator designs and circumvents the currently strained ventilator component supply chain. As an example, researchers have substituted more premium components such as proportional valves and flow sensors with inexpensive pinch valves^20,22^ and differential pressure sensors, respectively.^24^ These prototypes still require numerous components, however, which may not be suitable for mass production and rapid deployment in a short period of time.

Because the primary design challenge still remains, several universities have been pursuing further simplified solutions by automating a standard manual BVM (e.g., AmbuBag). A list of the representative BVM-based ventilator projects with relevant specifications and features is summarized in **Table 1**, including MIT’s E-Vent project, which provides open access to the detailed design files. BVM-based ventilators have the distinct advantages of availability, minimal number of components, simple mechanism, low cost, and the capability for rapid deployment. The majority of currently available BVM-based designs, however, all rely on the machining or 3D printing and assembly of a series of mechanical transmission components. Machined parts can be challenging to mass produce during lockdown periods, while 3D-printed parts may experience limited lifespan and eventually catastrophic failure. In addition, these mechanically driven systems require manual loading and alignment of the AmbuBag within the machine arms to achieve optimal compression of the bag to achieve adequate oxygenation and ventilation. Alternatively, OxVent, a joint research effort from University of Oxford and King’s College London, takes a different route, using pneumatic control and compressed air to compress the AmbuBag and deliver a breath. The OxVent design seals the AmbuBag inside of the chamber permanently, which is problematic in clinical situations in which manual bagging is required, because there is no way to switch the automated bagging back to a manual mode if a control failure occurs. One potential advantage of permanently sealing the chamber, however, is that it could offer higher reliability (e.g., avoid leaking) for the pressurized compartment.^25^

**Table 1.** A List of Representative Projects on BVM-Based Ventilators for COVID-19.

Project	Developers	Tidal Volume (mL)	Respiratory Rate (bpm)	I:E Ratio	Features and Notes	Links and Sources
**AmboVent**	Innovators in Israel	N/A; 30–100% of full squeezing capacity	6–24	N/A	Open-source; uses simple robotic arm for compression; includes safety features like 2 h battery backup, and cutoff mechanism for high resistance and high pressure; PIP from 30 to 70 cmH_2_O.	https://ambovent.org
**ApolloBVM**	Rice University	300–650	5–30	1:2 to 1:5	Open-source; dual rack-and-pinion mechanical design; controls are clinician-designed with adult, child, and pediatric settings; capable to operate for 24 h continuously.	http://oedk.rice.edu/apollobvm/
Coventor*	University of Minnesota and Boston Scientific Corporation	N/A; 4–8 mL/kg of predicted body weight	N/A, max at 30	1:1	Open-source; first-of-kind BVM-based ventilator authorized by FDA EUA; uses a slider-crank mechanism; PEEP up to 20 cmH_2_O; pressure relief valve of 40 cmH_2_O.	https://med.umn.edu/covid19Ventilator
**Emergency Ventilator (E-Vent)**	MIT	200–800	6–40	1:1 to 1:4	Open-source; uses a motor-driven cam mechanism to squeeze AmbuBag; assist control mode to detect pressure dip from 2 to 7 cmH_2_O; PIP at 40 cmH_2_O, plateau pressure at 30 cmH_2_O, and PEEP from 5 to 15 cmH_2_O. The listed specifications and functions are the targeted goals that might not have been fully validated.	https://e-vent.mit.edu/
**OpenVent-Bristol**	Innovators in UK	N/A; max at 800	10–30	1:1 to 1:3	Open-source; motorized-arm-enabled squeezing; PIP limited to 45 cmH_2_O. Specifications are extracted from an open-source online DIY tutorial.	https://openventbristol.co.uk/
**OxyGEN**	Protofy	450–650	0–32	1:1 to 1:5	Camshaft and lever-enabled squeezing; received AEMPS approval to be used in clinical studies; standard I:E ratio at 1:2, other ratios without guarantee of delivery precision; PIP limited to 40 cmH_2_O by a safety valve, and PEEP from 10 to 30 cmH_2_O; provides selective cams based on the required tidal volume and I:E ratio. Specifications are extracted from its user manual.	https://www.oxygen.protofy.xyz/
**OxVent**	University of Oxford and King’s College London	250–600	10–30	1:1 to 1:3	Pneumatic compression mechanism; no moving parts; PEEP from 5 to 20 cmH_2_O, PIP from 15 to 35 cmH_2_O; AmbuBag was sealed in a compression chamber.	https://oxvent.org/
PREVENT*	PVA	200–700	8–25	1:1 to 1:3	Uses two actuating arms for squeezing; PEEP from 5 to 20 cmH_2_O, and PIP from 10 to 40 cmH_2_O; alarms for pressure greater than PIP or lower than 2 cmH_2_O for 5 s.	https://contact.pva.net/prevent/
Spiro Wave*	Spiro Devices LLC	200–800	10–35	1:1 to 1:4	Inspired by MIT E-Vent project, with a similar working mechanism; PEEP up to 25 cmH_2_O; backup power supply lasts for 10 min.	https://ventilatorresponse.com/
Umbulizer*	Umbulizer LLC	0–700	1–30	1:0 to 1:3	Uses a motorized paddle for squeezing; PIP from 0 to 50 cmH_2_O; inspiratory time from 0.5 to 3 s; specifications are extracted from a relevant patent.^28^	https://www.umbulizer.com/
**AmbuBox**	MiNI Lab at University of California, Davis	250–800	10–30	N/A	Open-source; no moving parts; bidirectional sealing as a fail-safe feature; PEEP from 5 to 20 cmH_2_O, and PIP from 15 to 40 cmH_2_O; avoids alignment and slipping issues; independent and modular design; smallest footprint and lightweight design.	https://mini.ucdavis.edu/

AEMPS: Spanish Agency for Medicines and Health Products; bpm: breaths per minute; BVM: bag valve mask; COVID-19: coronavirus disease 2019; DIY: do-it-yourself; EUA: Emergency Use Authorization; FDA: US Food and Drug Administration; I:E ratio: inspiratory to expiratory ratio; MIT: Massachusetts Institute of Technology; N/A: not available or not reported; PEEP: positive end-expiratory pressure; PIP: peak inspiratory pressure.

* Devices approved by FDA EUA.

In this study, we propose and demonstrate a low-cost clinically viable ventilator design, AmbuBox, using a controllable pneumatic enclosure and standard manual resuscitators that are readily available, which can be rapidly deployed during mass-casualty and pandemic situations with a minimal set of components to manufacture and assemble. The current AmbuBox ventilator is arguably the simplest and smallest ventilator, with a modular and independent design and bidirectional sealing for the AmbuBag; it is assembled with laser-cut and off-the-shelf parts, while maintaining high accuracy and long-lasting stability. We have demonstrated the AmbuBox for adjustable control of tidal volume (TV) from 250 to 800 mL within 10%, respiratory rate (RR) from 10 to 30 bpm (breaths per minute) within 2 bpm, and pressure monitoring within 2 cmH_2_O. The positive end-expiratory pressure (PEEP) can be adjusted from 5 to 20 cmH_2_O, and the peak inspiratory pressure (PIP) limit can be set from 15 to 40 cmH_2_O in addition to the relevant alarms for high and low pressures. The AmbuBox ventilator is a viable ventilator design to fill the gap between the conventional ventilators and AmbuBags, by retaining the critical functions of a ventilator for emergent use during the current COVID-19 pandemic and in mass-casualty situations, as well as emergent clinical use in low-resource settings.

## Materials and Methods

**Figure 1.**
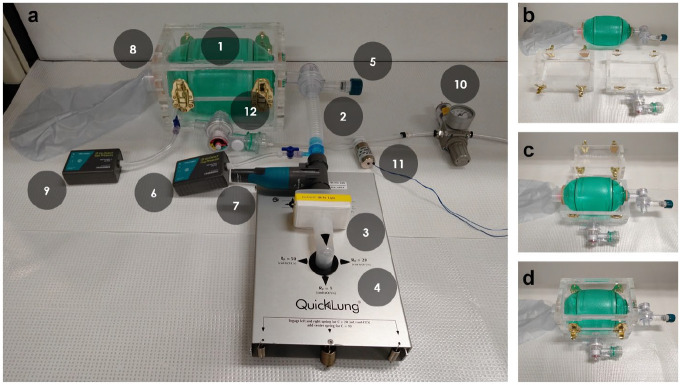
Overview of the testing system and assembly of AmbuBox. (**a**) Photo of the testing system, with key components labeled; and (**b–d**) the bidirectional assembly of AmbuBag into the AmbuBox chamber.

**Figure 1a** shows the testing system of AmbuBox with key components labeled. The AmbuBag (1, AirFlow manual resuscitator adult size, SunMed Medical, Marlton, NJ) was connected with an extension tubing (2) and a HEPA filter (3, ISO-Gard HEPA Light, Teleflex, Morrisville, NC) into a test lung (4, QuickLung Breather, IngMar Medical, Pittsburgh, PA). The exhausted air from the test lung was drained from a manually adjustable PEEP valve (5, VP700, SunMed Medical) connected with the AmbuBag. A pressure sensor (6, Go Direct Gas Pressure Sensor, Vernier, Beaverton, OR) and a spirometer (7, Go Direct Spirometer, Vernier) were connected inline between the extension tubing and the filter with an adapter that was originally used for oxygen enrichment in CPAP machines. The AmbuBag was bidirectionally sealed in a laser-cut acrylic AmbuBox chamber (8). An additional Vernier pressure sensor (9) was connected to monitor the chamber pressure. Compressed air from the wall was connected to a pressure regulator (10, SAW2000M-N02BG, PneumaticPlus, Torrance, CA) and then attached to a three-way solenoid valve (11, Masterflex 3-way solenoid pinch valve, Cole-Parmer, Vernon Hills, IL). One end of the solenoid valve was connected with atmosphere, while the other end was connected to the chamber with an adapter and a one-way valve (12), which was detached from the AmbuBag. A microcontroller unit (MCU) with adjustment knobs and an accessory LCD display were used to control the solenoid valve and monitor the conditions in the simulated patient airway and the AmbuBox chamber.

**Figure 1b–1d** shows the process of the bidirectional sealing of AmbuBag into the acrylic chamber. As shown in **Figure 1b**, the AmbuBox chamber was made of two components: an upper cap and a lower cap. Each of the two caps was prepared by laser ablation (Epilog Mini 18/24 laser system, Epilog Laser, Golden, CO) of five pieces of 0.5-inch-thick acrylic sheets, with the interlock structures designed for assisted and simplified assembly. As an alternative to using a standard laser cutter to make these components, in resource-deprived situations one could alternatively use other, easier-to-access tools such as a power saw or hand saw. The caps were assembled and bonded using acrylic cement (cat. no. 10315, IPS Corporation, Compton, CA). Each of the caps was designed with curved edges at the ends to best fit the contour of the AmbuBag. Four clasp clamps were installed to hold and seal the two caps tightly with rubber bands attached to both of the two heads of the AmbuBag to assist sealing, while reserving the function for convenient separation of the caps to quickly access the AmbuBag in situations of emergency or system failure.

In detail, the electronic control unit consists of an MCU module (Arduino Mega R3, Arduino, www.arduino.cc), a 1602 LCD display, two adjustment knobs, and accessories such as solid-state relay (KF0602D, Kyotto, Kytech Electronics, Shenzhen, China) and a power supply adapter (Chanzon, Shenzhen, China). In the first stage, the pressure sensors and spirometer from Vernier were used for the characterization of the AmbuBox ventilator, due to their high precision and convenient graphical user interface (GUI) for data display, in addition to their capability for Bluetooth-enabled wireless data transmission. One snapshot of the collected data in real time is shown in **Supplementary Figure S1**. For such purposes, the AmbuBox ventilator was working under the inspiratory duration-controlled mode, and the MCU was used for the adjustment of opening and closure duration of the solenoid valve. Specifically, inflation durations from 0 to 3 s with adjustment of 0.01 s were generated, and respiratory rates ranging from 10 to 30 bpm were produced. The PEEP and PIP pressures were monitored through Vernier Graphical Analysis 4, a cross-platform program. In the second stage, the AmbuBox ventilator was working under tidal volume–controlled mode. A disposable commercially available ventilator flow sensor (PN 281637 Medical Flow Sensor, Hamilton Medical, Reno, NV) was connected into the breath circuit to replace the Vernier spirometer. The pressure drop across the flow sensor was monitored using a differential pressure sensor (SDP 816, Sensirion AG, Staefa, Switzerland), which was calibrated to determine the flowrate. In addition, the inspired or expired volume across the flow sensor was calculated by integrating the transient flowrates within the MCU. An algorithm was implemented to deactivate the solenoid valve once the cumulative volume reached the preset tidal volume threshold. In addition, two pressure sensors (MPX5050DP, NXP, Eindhoven, the Netherlands) were installed to replace the two Vernier pressure sensors for the real-time monitoring of the simulated patient (QuickLung, IngMar Medical) inside the breath circuit and the AmbuBox chamber, respectively. Pressure safety alarms were enabled by the MCU to generate buzzer alarms for pressures higher than 40 cmH_2_O or lower than 15 cmH_2_O. When the alarms are activated, they will continue until a clinician intervenes and adjusts the relevant parameters to return the PIP to within the safe range. Two knobs were used to adjust the tidal volume and respiratory rate, respectively. An LCD display was used to show the PEEP and PIP pressure in real time, as well as the tidal volume and respiratory rate. A block diagram of the electrical connections is shown in **Supplementary Figure S2**. It is worth noting that because the aforementioned solenoid pinch valve has a maximum working pressure of 20 psi, it was used for low-input air pressures, while a conventional solenoid valve (PV0178, TEMCo Industrial, Fremont, CA) could be used for input pressures higher than 20 psi.

**Supplementary Table S1** summarizes the cost breakdown of the components needed to fabricate the AmbuBox, totaling less than $300. Biocompatibility has been taken into consideration in the selection of materials and parts of AmbuBox to facilitate the future application of FDA EUA, in which the components of AmbuBox that connected directly to the patient’s breath circuits are all in medical grades. In particular, such components include a medical AmbuBag with accessory PEEP valve, an extension tubing typically used for medical CPAP machines, a ventilator HEPA filter, two adapters typically used in medical CPAP machines, a disposable flow sensor used for medical ventilators, and a pressure sensor. Details about the model numbers and vendor sources are listed in **Supplementary Table S1**. Given that its components either are off-the-shelf or can be conveniently laser cut, the AmbuBox can be mass produced locally in a short period of time.

## Results and Discussion

In this part, we first described the operating principles of AmbuBox. Next, the minimal functions of a clinically viable ventilator were discussed, and we identified that tidal volume and respiratory rate are the two most critical parameters clinicians emphasize. Based on the operating principles of the AmbuBox, we then investigated the inflation/deflation and inspiration/expiration dynamics of the AmbuBox, by real-time monitoring the pressure and flow inside both the AmbuBox chamber and the simulated-patient breath circuit, to correlate the dynamics of AmbuBox with the two critical parameters aforementioned. Third, we conducted a thorough parametric study of the dependence of tidal volumes and respiratory rates on the various control parameters and patient conditions, such as the inflation duration, and the compliance and resistance of the patient lung, as tabularized in **Supplementary Table S2**. Furthermore, we demonstrated the working performance of the AmbuBox ventilation system in the real-time adjustment of tidal volume, respiratory rate, PEEP pressure, and long-term stability.

### Operating Principles

**Figure 2.**
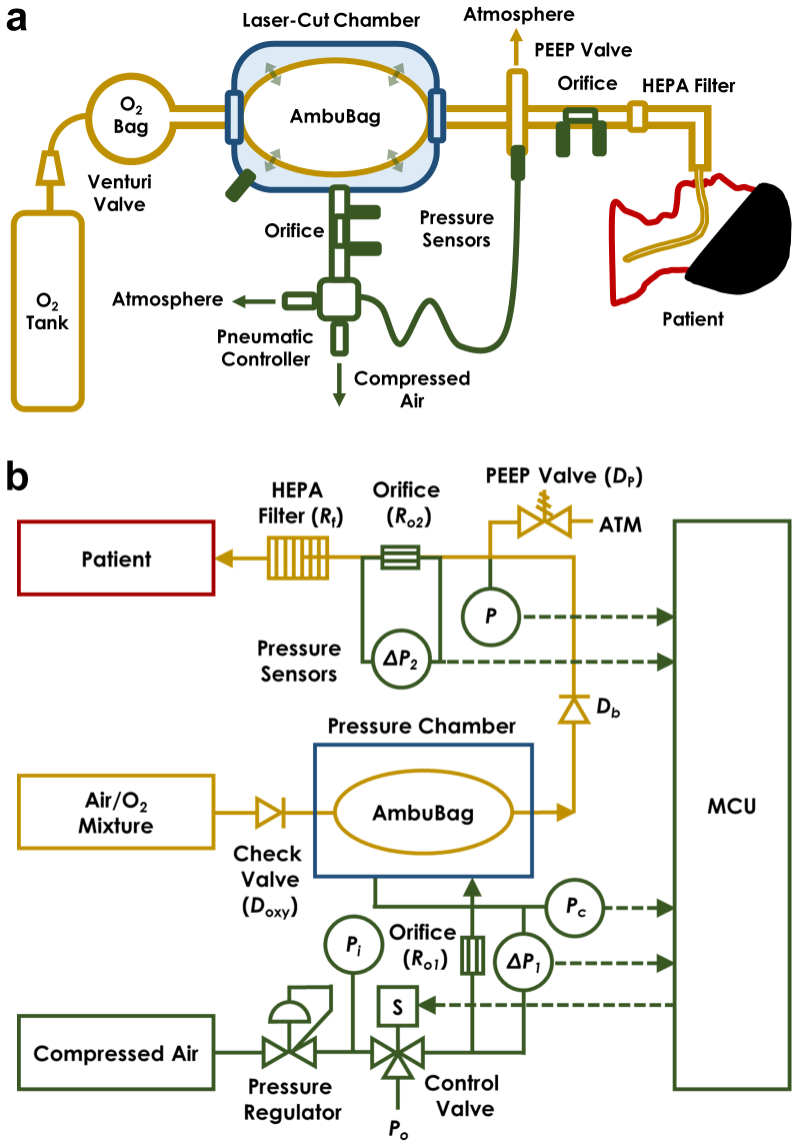
Concept of AmbuBox ventilator. (**a**) Schematic illustration of the AmbuBox ventilator; and (**b**) system-level block diagram of the control system, including the pneumatic valve control and signal transmission from the sensors to the microcontroller unit (MCU).

The AmbuBox ventilator functions through an automated pneumatically driven squeezing and releasing of an AmbuBag. Specifically, the AmbuBox consists of three components (**Fig. 2a**): (1) a standard AmbuBag-type manual resuscitator with its accessories (yellow-colored), (2) a laser-cut chamber (blue-colored) with airtight and bidirectional sealing to the AmbuBag, and (3) a pneumatic control unit with solenoid valves and pressure- and flow-sensing feedback (green-colored). The AmbuBag can be placed bidirectionally inside the AmbuBox, where it is squeezed by pressurizing the AmbuBox via a positive-pressure air source (e.g., compressed gas line, gas cylinder, or air pump). The solenoid valve allows for precise control of the duration of inflation and deflation of the AmbuBox. As a result, the AmbuBox pushes oxygenated air from the AmbuBag through the extension tubing into an inspired breath for the patient. The expired air from the patient is exhausted from a standard adjustable PEEP valve, while passing through a HEPA filter to capture aerosols from the patient, protecting health care workers. A gauge pressure transducer and a differential pressure-based flowrate-detecting orifice are connected along the breathing circuit of the patient to monitor any aberrancies in the airway pressure and flowrate. Similarly, a gauge pressure transducer and a flow sensor are installed between the pneumatic controller and the AmbuBox chamber to monitor the pressure and flowrate during the inflation and deflation of the AmbuBox chamber. A standard MCU with a simple user interface allows adjustment of respiratory rate (RR) and tidal volume (TV). In addition, PIP and PEEP are both displayed. The PEEP can be manually set via the adjustable PEEP valve, and the alarms for high and low pressures are included in the MCU. In addition, the fraction of inspired oxygen (FiO_2_) can be regulated with a Venturi flow valve. **Figure 2b** shows a system-level block diagram of the pneumatic valve control and signal transmission from the sensors to the user interface.

**Figure 3.**
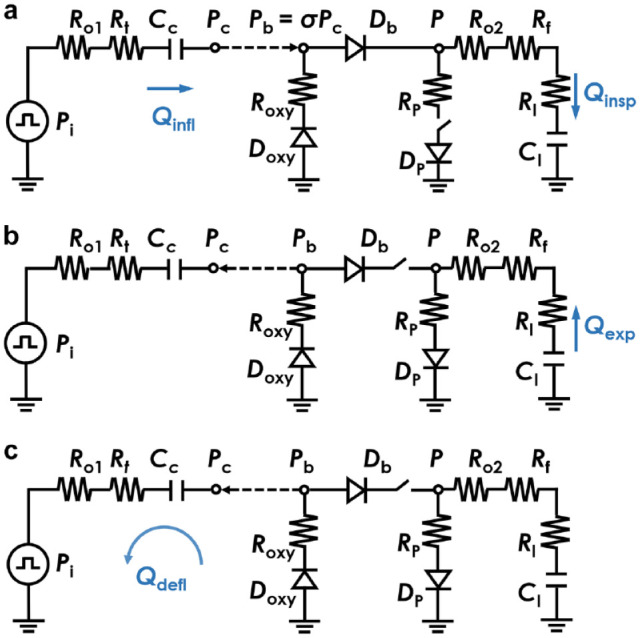
Pneumatic circuit model of three processes in AmbuBox. (**a**) Inflation of the AmbuBox chamber and inspiration of the patient; (**b**) expiration of the patient; and (**c**) deflation of the AmbuBox chamber.

To model the functionality of the proposed AmbuBox ventilator, a pneumatic-electronic analogical circuit has been developed, as illustrated in **Figure 3**. Briefly, the operation of the AmbuBox ventilator can be separated into three processes, namely the inflation of the AmbuBox chamber (inspiration of the patient), the expiration of the patient, and the deflation of the chamber. It is worth mentioning that all three processes contribute to the setting of respiratory rate and tidal volume, two of the most critical parameters for a ventilator design. In the initial process, as shown in **Figure 3a**, high-pressure $\left(P_{i}\right)$ compressed air passes through the flow sensor $\left(R_{o1}\right)$ and relevant tubing $(R_{t}),$ and it inflates the AmbuBox chamber $\left(C_{c}\right)$ and increases the chamber pressure $(P_{c}).$ As a result, the buildup of the surrounding pressure compresses the AmbuBag, and the pressure inside the AmbuBag $\left(P_{b}\right)$ increases correspondingly. The increase in AmbuBag pressure opens the one-way valve $\left(D_{b}\right)$ at the outlet head, and it drives the air–oxygen mixture inside the bag into the breath circuit of the patient, passing through a flow sensor $\left(R_{o2}\right)$ and a HEPA filter $(R_{f}).$ This generates a positive pressure in the lung of the patient (with lung resistance of $R_{l}$ and compliance of $C_{l}).$ Both the airway pressure and inspiratory flowrate $\left(Q_{insp}\right)$ along the breath circuit are monitored as indicators and feedback controls of the lung pressure, inspiratory flowrate, and tidal volume. In addition, the pressure inside the AmbuBox chamber $\left(P_{c}\right)$ and the inflation flowrate $\left(Q_{infl}\right)$ are also monitored. During the second process, namely the expiration as shown in **Figure 3b**, the one-way valve $\left(D_{b}\right)$ at the outlet of the AmbuBag is blocked, and the exhaled CO_2_ from the patient is released into the atmosphere through the PEEP valve $(D_{p}).$ Similarly, sensors along the breath circuit monitor the pressure and expiratory flowrate $\left(Q_{exp}\right)$ during the expiration process. In the third process, as shown in **Figure 3c**, the AmbuBox chamber deflates by switching the solenoid valve to connect the chamber with the ambient pressure. As the chamber pressure reduces, the compressed AmbuBag tends to return to its original shape, which generates a negative pressure inside the bag. Subsequently, the one-way valve $\left(D_{oxy}\right)$ opens at the inlet head of the AmbuBag, and that allows the air–oxygen mixture to fill up the AmbuBag. The AmbuBox chamber pressure during the deflation and the deflation rate $\left(Q_{defl}\right)$ can be monitored as indicators of the status of the AmbuBag to determine whether the bag has been fully inflated again.

### Minimal Functions of a Clinically Viable Ventilator

By referring to the emergency use ventilator (EUV) guideline^26^ and literature, in addition to the clinical demand at University of California Davis Medical Center (UCDMC), the following requirements of the AmbuBox ventilator have been identified to make a minimal viable product (MVP) for emergent clinical settings. The minimal requirements include (1) two controls: respiratory rate from 10 to 30 bpm within 2 bpm, and tidal volume from 250 to 800 mL within 10%; (2) adjust and monitor two pressures within 2 cmH_2_O (PEEP pressure from 5 to 20 cmH_2_O, and PIP pressure from 15 to 40 cmH_2_O); and (3) alarms for pressures that are too high or too low (with safety presets). It is worth noting that the proposed ventilator is aimed to be used for the resuscitation of sedated patients. In such scenarios, only periodic ventilation is required.

### Dynamics of AmbuBox

**Figure 4.**
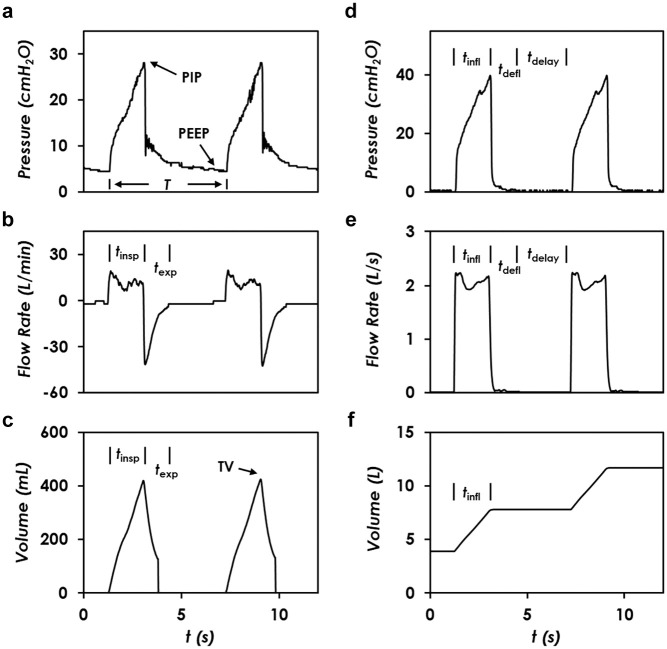
Real-time monitoring of the pressure and flow conditions in patient airway and AmbuBox chamber. Readings from the patient’s breath circuit, including (**a**) airway pressure, (**b**) inspiratory flowrate, and (**c**) cumulative volume entering the patient lung. Readings from the AmbuBox chamber, including (**d**) chamber pressure, (**e**) inflation rate, and (**f**) volume of compressed air entering the AmbuBox chamber. The testing conditions are as follows: compliance (C) = 20 mL/cmH_2_O, resistance (R) = 20 cmH_2_O/(L/s), positive end-expiratory pressure (PEEP) = 5 cmH_2_O, input pressure $\left(P_{i}\right)$ = 15 psi, respiratory rate (RR) = 10 bpm, and duration of inflation $\left(t_{infl}\right)$ = 1.8 s.

To further clarify the correlation between the pneumatic controls of the three processes and the resultant respiratory rate and tidal volume, the typical transient pressure, the flowrate, and the cumulative volume along the breath circuit of the simulated patient and the AmbuBox chamber are plotted in **Figure 4**. The testing conditions and preset parameters are as follows: Compliance C is 20 mL/cmH_2_O, resistance R is 20 cmH_2_O/(L/s), PEEP pressure is 5 cmH_2_O, input compressed air pressure $P_{i}$ is 15 psi, respiratory rate RR is 10 bpm (the corresponding breath period is 6 s), and duration of inflation $t_{infl}$ is 1.8 s. As labeled in **Figure 4a**, the PEEP pressure is measured around 4.7 cmH_2_O, close to the preset PEEP pressure value, yet with a slight inaccuracy caused by the PEEP valve itself, but still within the acceptable precision (2 cmH_2_O). PIP is measured around 28 cmH_2_O. Furthermore, the respiratory period T is measured to be 6 s. As shown in **Figure 4b**, the measured inspiratory duration $t_{insp}$ is 1.78 s, and the expiratory duration $t_{exp}$ is measured 1.28 s. The inspiratory rate $Q_{insp}$ ramps up to the maximum and forms a relatively constant plateau flowrate of approximately 12 L/min, during the process of inspiration. By contrast, the same amount of volume of inhaled air is exhaled out from the patient during the expiratory process. Expectedly, the experimentally observed expiratory flowrate has reached the maximum at the beginning of expiration and then gradually reduced to zero, following the trend of the pressure difference, which is consistent with the ramping-down trend of airway pressure, as illustrated in **Figure 4a**. The expiration duration $\left(t_{exp}\right)$ is mainly influenced by the airway pressure and the preset PEEP pressure, between which the difference drives the expiratory air flow. In addition, the pressure oscillation has been observed during the expiration process, as shown in **Figure 4a**, which is probably due to the membrane vibration inside the PEEP valve. The ventilated tidal volume is around 420 mL, as shown in **Figure 4c**. In addition, the inflation duration $t_{infl}$ in the AmbuBox chamber is 1.78 s, as shown in **Figure 4d**, identical to the inspiratory duration as expected, $t_{infl}=t_{insp}.$ Moreover, the deflation duration $t_{defl}$ and the time of delay $t_{delay}$ are measured at 0.82 s and 3.4 s, respectively, where the maximum chamber pressure has been found at around 40 cmH_2_O. As shown in **Figure 4e**, during the process of inflation, the rate of inflation $Q_{infl}$ reaches an approximately constant plateau at 2 L/s. Lastly, the amount of compressed air entering the chamber in each process of inflation is approximately 3.9 L, which is the step volume increment as shown in **Figure 4f**. Therefore, a standard J size medical cylinder of compressed air, which has a water capacity of 47.2 L at 137 bar,^27^ can continuously ventilate for approximately 83 min under the current testing conditions.

The detailed correlation of respiratory rate and tidal volume with the specifications of the AmbuBox are discussed below. As shown in **Figure 4a**, the respiratory rate (RR; in units of bpm) correlates with the breath period (T, in units of second) of each breathing cycle as RR=60/T. In addition, the period can be separated into three components: the duration of inspiration/inflation $(t_{insp}=t_{infl}),$ the duration of deflation $(t_{defl}),$ and the duration of delay $\left(t_{delay}\right)$ between adjacent cycles. In other words, respiratory rate correlates with the three durations as $RR=60/\left(t_{infl}+t_{defl}+t_{delay}\right).$ It is worth mentioning that both $t_{infl}$ and $t_{delay}$ can be actively controlled by adjusting the opening/closure duration of the solenoid valve or the input inflation pressure. In contrast, $t_{defl}$ is passively determined because it relies on the intrinsic deformability of the AmbuBag. As a result, $t_{defl}$ can be a limiting factor for the control of respiratory rate. That being said, several systematic improvements can be made to shorten $t_{defl},$ for instance (1) reducing the dead volume inside the AmbuBox chamber $(C_{c}),$ (2) lowering the flow resistance of the connecting tubing $(R_{t}),$ or (3) connecting the AmbuBox chamber with a vacuum pressure source to speed up the deflation rate. It is good practice to measure $t_{defl}$ under various working conditions so that we know the theoretical maximum respiratory rate, which is approximately $1/t_{defl},$ assuming that the time of delay is zero and the duration of inflation is significantly short. The tidal volume, however, is mainly dictated by the inflation/inspiration process, following $TV=\overset{t_{insp}}{\underset{0}{\int }}Q_{insp}dt.$ Once the inflation pressure $\left(P_{i}\right)$ and flow resistances are fixed, the duration of inspiration $t_{insp}$ (same as $t_{infl}),$ would govern the tidal volume, as illustrated in **Figure 4c**. It is worth noting that a higher tidal volume results in a greater deformation of the AmbuBag, and therefore, the corresponding deflation duration $t_{defl}$ will rise, along with a reduced RR accordingly. This presents a tradeoff of the high tidal volume and the high respiratory rate.

### Parametric Studies of Tidal Volume and Respiratory Rate

**Figure 5.**
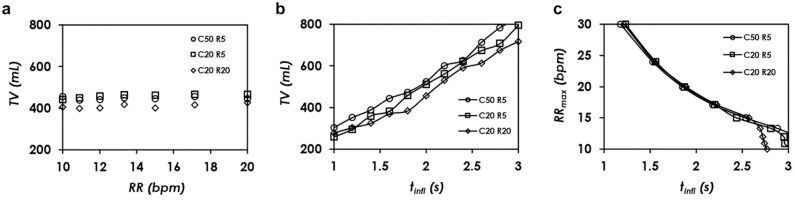
Parametric studies of the AmbuBox ventilator. (**a**) The tidal volume remains a constant as the respiratory rate changes from 10 to 20 breaths per minute (bpm), under the testing conditions of compliance (C) from 20 to 50 mL/cmH_2_O, resistance (R) from 5 to 20 cmH_2_O/(L/s), peak end expiratory pressure (PEEP) = 5 cmH_2_O, input pressure $\left(P_{i}\right)$ = 15 psi, and duration of inflation $\left(t_{infl}\right)$ = 1.8 s. (**b**) Tidal volume is dependent on inspiratory duration, under the testing conditions of C from 20 to 50 mL/cmH_2_O, R from 5 to 20 cmH_2_O/(L/s), PEEP = 5 cmH_2_O, $P_{i}$ = 15 psi, and respiratory rate (RR) = 10 bpm. (**c**) The maximal respiratory rate is dependent on inspiratory duration, under the testing conditions of C from 20 to 50 mL/cmH_2_O, R from 5 to 20 cmH_2_O/(L/s), PEEP = 5 cmH_2_O, and $P_{i}$ = 15 psi.

First, we investigated how tidal volume varied at different respiratory rates. As shown in **Figure 5a**, under the testing conditions of C = 20 mL/cmH_2_O, R = 20 cmH_2_O/(L/s), PEEP = 5 cmH_2_O, $P_{i}$ = 15 psi, and $t_{infl}$ = 1.8 s, the tidal volume was kept at a constant of 410 mL from 10 bpm to 20 bpm. When the respiratory rate was higher than 20 bpm, the AmbuBag did not fully return to its original shape. This maximum rate is limited by the speed of releasing of the bag, which is passively controlled as aforementioned. Respiratory rate lower than 10 bpm was not studied due to the irrelevance for the targeted COVID-19 ventilation applications. These results indicate that the two parameters under study, namely the tidal volume and respiratory rate, can be decoupled from each other within a confined range of respiratory rate. In addition, as shown in **Figure 5a**, increasing the compliance to 50 mL/cmH_2_O resulted in a larger tidal volume of approximately 440 mL, while reducing the resistance to 5 cmH_2_O/(L/s) also increased the tidal volume, to reach approximately 460 mL. Next, we studied how the adjustable inflation duration $t_{infl}$ affected the tidal volume and respiratory rate. As shown in **Figure 5b**, the tidal volume was measured by increasing the inflation duration from 1 s to 3 s in an increment of 0.2 s. Under the testing conditions of C = 20 mL/cmH_2_O, R = 20 cmH_2_O/(L/s), PEEP = 5 cmH_2_O, $P_{i}$ = 15 psi, and RR = 10 bpm, it was found that the tidal volume increased approximately linearly from 280 to 720 mL. Further increasing the inflation duration resulted in PIP pressure higher than 40 cmH_2_O, a safety preset pressure. Inflation duration shorter than 1 s resulted in tidal volume smaller than 250 mL, irrelevant for COVID-19 ventilation applications. In addition, increasing the compliance to 50 mL/cmH_2_O or reducing the resistance to 5 cmH_2_O/(L/s) both resulted in slightly larger tidal volume, covering a linear increase from 300 to 830 mL and 260 to 800 mL, respectively, during the duration range from 1 s to 3 s. Lastly, the dependence of the maximum respiratory rate $RR_{max}$ on the inflation duration was investigated, with results shown in **Figure 5c**. Two criteria were used to determine $RR_{max}:$ (1) PIP pressure smaller than 40 cmH_2_O; and (2) AmbuBag fully inflates back to its original shape after each cycle of breath. During the testing process, we first set the respiratory rate to be from 10 to 30 bpm, and then the inflation duration was increased by increments of 0.01 s from zero, until either of the two criteria was matched. As shown in **Figure 5c**, under the testing conditions of C = 20 mL/cmH_2_O, R = 20 cmH_2_O/(L/s), PEEP = 5 cmH_2_O, and $P_{i}$ = 15 psi, the maximum respiratory rate of 30 bpm corresponds to an inflation duration of 1.22 s, and the maximum respiratory rate of 10 bpm corresponds to an inflation duration of 2.77 s. The maximum respiratory rate decreases as the inflation duration increases. As aforementioned, longer inflation duration leads to more severe deformation of the AmbuBag, and therefore it takes longer to deflate, resulting in lower respiratory rate. It is worth noting that the last four data points, namely when inflation duration is longer than 2.59 s, are mainly dictated by the first criterion (PIP lower than 40 cmH_2_O), while the data points with shorter inflation duration are dictated by the second criterion (AmbuBag fully inflates back to its original shape). Increasing the compliance or reducing the resistance results in similar trends, except that both of them are mainly governed by the second criterion, as indicated in **Figure 5c**. As we would expect, higher compliance or smaller resistance both help reduce the airway pressure, and as a result, the first criterion would be more difficult to reach. Synchronically looking at **Figure 5b** and **Figure 5c**, the AmbuBox is capable of reaching a tidal volume of up to 350 mL at a respiratory rate of 30 bpm, and a tidal volume of up to 830 mL at a respiratory rate of 10 bpm. In addition, the inflation duration can be conveniently adjusted to cover our targeted range of respiratory rate and tidal volume.

### Demonstration

**Figure 6.**
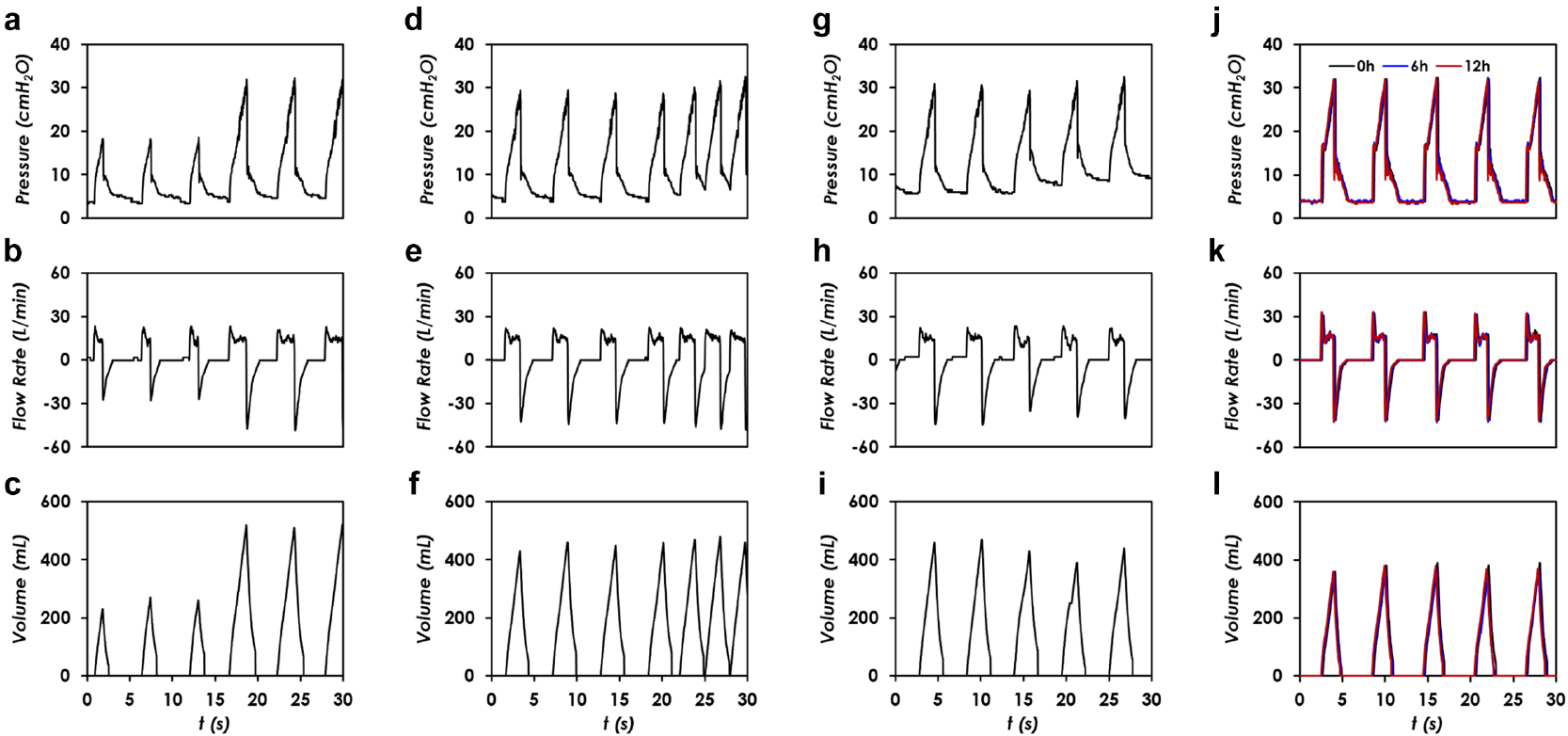
Demonstration of AmbuBox ventilator. (**a–c**) Real-time adjustment of tidal volume, under the testing conditions of compliance (C) = 20 mL/cmH_2_O, resistance (R) = 20 cmH_2_O/(L/s), peak end expiratory pressure (PEEP) = 5 cmH_2_O, input pressure $\left(P_{i}\right)$ = 15 psi, and respiratory rate (RR) = 10 bpm; (**d–f**) adjustment of respiratory rate, under the testing conditions of C = 20 mL/cmH_2_O, R = 20 cmH_2_O/(L/s), PEEP = 5 cmH_2_O, $P_{i}$ = 15 psi, and duration of inflation $\left(t_{infl}\right)$ = 1.8 s; (**g–i**) adjustment of PEEP pressure, under the testing conditions of C = 20 mL/cmH_2_O, R = 20 cmH_2_O/(L/s), $P_{i}$ = 15 psi, RR = 10 bpm, and $t_{infl}$ = 1.8 s; and (**j–l**) long-term stability test with a brand-new AmbuBag, under the testing conditions of C = 20 mL/cmH_2_O, R = 20 cmH_2_O/(L/s), PEEP = 5 cmH_2_O, $P_{i}$ = 15 psi, RR = 10 bpm, and $t_{infl}$ = 1.5 s.

Following the thorough characterization of AmbuBox, we demonstrated the real-time adjustment of tidal volume, respiratory rate, and PEEP pressure using an AmbuBox, together with its long-term stability, as shown in **Figure 6**. First, the tidal volume can be conveniently adjusted by increasing the inflation duration. As shown in **Figure 6a–6c**, we increased the inflation duration from 1 s to 2 s, and the corresponding tidal volume was raised up from 270 mL to 520 mL, under the testing conditions of C = 20 mL/cmH_2_O, R = 20 cmH_2_O/(L/s), PEEP = 5 cmH_2_O, $P_{i}$ = 15 psi, and RR = 10 bpm. The corresponding PIP was increased from 18 to 32 cmH_2_O, while the inspiratory flowrate was approximately kept the same at 13 L/min. We also demonstrated the real-time adjustment of respiratory rate from 10 bpm to 20 bpm, as shown in **Figure 6d–6f**, under the testing conditions of C = 20 mL/cmH_2_O, R = 20 cmH_2_O/(L/s), PEEP = 5 cmH_2_O, $P_{i}$ = 15 psi, and $t_{infl}$ = 1.8 s. The tidal volume remained the same at around 450 mL. The PIP pressure increased from 29 to 32 cmH_2_O, and the stable inspiratory flowrate remained the same at around 15 L/min. In **Figure 6g–6i**, we showed the capability to adjust the PEEP, under the testing conditions of C = 20 mL/cmH_2_O, R = 20 cmH_2_O/(L/s), $P_{i}$ = 15 psi, RR = 10 bpm, and $t_{infl}$ = 1.8 s. We adjusted the PEEP from 5 to 10 cmH_2_O. As shown in **Figure 6g**, the measured PEEP was increased from 5.7 cmH_2_O to approximately 9.2 cmH_2_O. The PIP increased slightly from 29 to 32 cmH_2_O, and the corresponding inspiratory plateau flowrate slightly reduced from 15 L/min to 13 L/min. In addition, the tidal volume decreased slightly from 460 mL to 410 mL.

Lastly, we tested the long-term stability and consistency of the AmbuBox ventilator, as shown in **Figure 6j–6l**, under the testing conditions of C = 20 mL/cmH_2_O, R = 20 cmH_2_O/(L/s), PEEP = 5 cmH_2_O, $P_{i}$ = 15 psi, RR = 10 bpm, and $t_{infl}$ = 1.5 s. The proposed AmbuBox system is intended to be primarily used for emergent or ambulatory use, which typically lasts for a few hours. We therefore conducted a continuous 12 h test, and the corresponding waveforms of the original signals, 6 h and 12 h signals, were collected and plotted on the same figure for comparison. It is worth noting that because a brand-new AmbuBag was used in the long-term stability test, the corresponding PIP and tidal volumes were slightly altered. As can be seen, all the three waveforms in the two tests overlapped with the initial signal, which proved that the AmbuBox was capable of generating consistent and stable ventilation.

In this article, we have presented a clinically viable and rapidly deployable low-cost ventilator, AmbuBox, for emergent care during the COVID-19 pandemic. The AmbuBox uses pneumatic control of compressed air to automate the bagging of a manual resuscitator, or BVM (AmbuBag). A pneumatic-electronic analogy model has been proposed to elucidate the dynamics of three processes during ventilation: the inflation of the AmbuBox chamber (inspiration of the simulated patient), the expiration of the simulated patient, and the deflation of the AmbuBox chamber. The AmbuBox device offers several distinct advantages over existing counterparts: (1) the simplest ventilator design with high control accuracy, with adjustable tidal volumes from 250 to 800 mL with an accuracy of 10%, respiratory rates from 10 to 30 bpm within adjustment of 2 bpm, adjustable PEEP pressure from 5 to 20 cmH_2_O, and the capability to monitor airway pressure with an accuracy of 2 cmH_2_O, in addition to alarms for pressures higher or lower than safety presets; (2) easy to use with a failure protection mechanism, enabled by the capability of bidirectional sealing; (3) long-term stability and reliability, owing to the unique pneumatically driven working principle that eliminates any moving parts; (4) a user-friendly design that excludes any issues observed in mechanical designs, such as alignment and slipping; (5) an independent and modular design with two parts (mechanical and electronic modules) working separately and enabling transitions from manual compression of the AmbuBag to an automated clinical grade ventilator; (6) the smallest footprint and most lightweight design, with all the parts capable of being packed into a standard toolbox kit; and (7) low cost and prompt manufacturability with standard laser cuts and off-the-shelf components for convenient assembly. Benefiting from its simple yet functional design, the AmbuBox uses a controllable pneumatic enclosure and a standard manual resuscitator and can be rapidly deployed with a minimal set of components to manufacture and assemble, enabling it to be a promising candidate for emergent use during the COVID-19 pandemic and mass-casualty events, as well as regular clinical use in low-resource settings.

## Research Data

4_Movie_S1_of_AmbuBox for AmbuBox: A Fast-Deployable Low-Cost Ventilator for COVID-19 Emergent Care Click here for additional data file. 4_Movie_S1_of_AmbuBox for AmbuBox: A Fast-Deployable Low-Cost Ventilator for COVID-19 Emergent Care by Zecong Fang, Andrew I. Li, Hongcheng Wang, Ruoyu Zhang, Xiyan Mai and Tingrui Pan in SLAS Technology This article is distributed under the terms of the Creative Commons Attribution 4.0 License ( http://www.creativecommons.org/licenses/by/4.0/) which permits any use, reproduction and distribution of the work without further permission provided the original work is attributed as specified on the SAGE and Open Access pages ( https://us.sagepub.com/en-us/nam/open-access-at-sage).

Supplemental_Material_for_AmbuBox_by_Fang,_et_al – Supplemental material for AmbuBox: A Fast-Deployable Low-Cost Ventilator for COVID-19 Emergent Care Click here for additional data file. Supplemental material, Supplemental_Material_for_AmbuBox_by_Fang,_et_al for AmbuBox: A Fast-Deployable Low-Cost Ventilator for COVID-19 Emergent Care by Zecong Fang, Andrew I. Li, Hongcheng Wang, Ruoyu Zhang, Xiyan Mai and Tingrui Pan in SLAS Technology
