# Supplementary material for: AmbuBox: A Fast-Deployable Low-Cost Ventilator for COVID-19 Emergent Care
Source: SLAS Technol. 2020 Sep 3;25(6):573–84. doi: 10.1177/2472630320953801 (PMC7472193; doi:10.1177/2472630320953801)
Supplement: Supplemental_Material_for_AmbuBox_by_Fang,_et_al – Supplemental material for AmbuBox: A Fast-Deployable Low-Cost Ventilator for COVID-19 Emergent Care [file Supplemental_Material_for_AmbuBox_by_Fang,_et_al.pdf]

Supplemental Material

AmbuBox: A Fast-deployable Low-cost Ventilator for COVID-19 Emergent Care

By Zecong Fang[1], Andrew I Li[2], Hongcheng Wang[1,3], Ruoyu Zhang[1,4], Xiyan Mai[1], and Tingrui Pan[1,4]

[1]Micro-Nano Innovations (MiNI) Laboratory, Department of Biomedical Engineering, University of California, Davis, CA, USA

[2]Department of Surgery, University of California Davis Medical Center (UCDMC), Sacramento, CA, USA

[3]School of Mechanical Engineering, Hangzhou Dianzi University, Hangzhou, China

[4]Department of Electrical and Computer Engineering, University of California, Davis, CA, USA

Correspondence: Tingrui Pan, Micro-Nano Innovations (MiNI) Laboratory, Department of Biomedical Engineering, University of California, Davis, CA, 95616, USA. E-mail: tingrui@ ucdavis.edu

Movie S1: Demonstration video of AmbuBox working under the testing conditions of C = 20mL/cmH_2O, R = 5cmH_2O/(L/s), PEEP = 5cmH_2O, P_i = 15psi, RR = 20bpm, and t_{infl} = 1.8s.

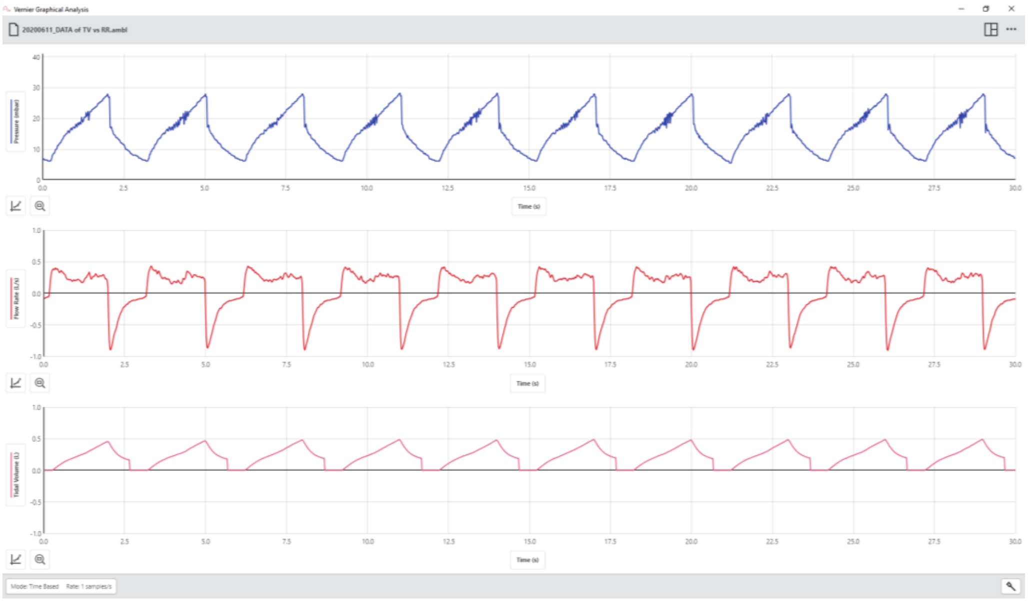

Figure S1. Real-time monitoring of pressure, flow rate and volume using Vernier pressure sensor and spirometer. Testing conditions are: C = 20mL/cmH_2O, R = 5cmH_2O/(L/s), PEEP = 5cmH_2O, P_i = 15psi, RR = 20bpm, and t_{infl} = 1.8s. The first row shows the real-time monitoring of the patient airway pressure, while the second row is the real-time inspiratory/expiratory rate and the third row is the cumulative volume.

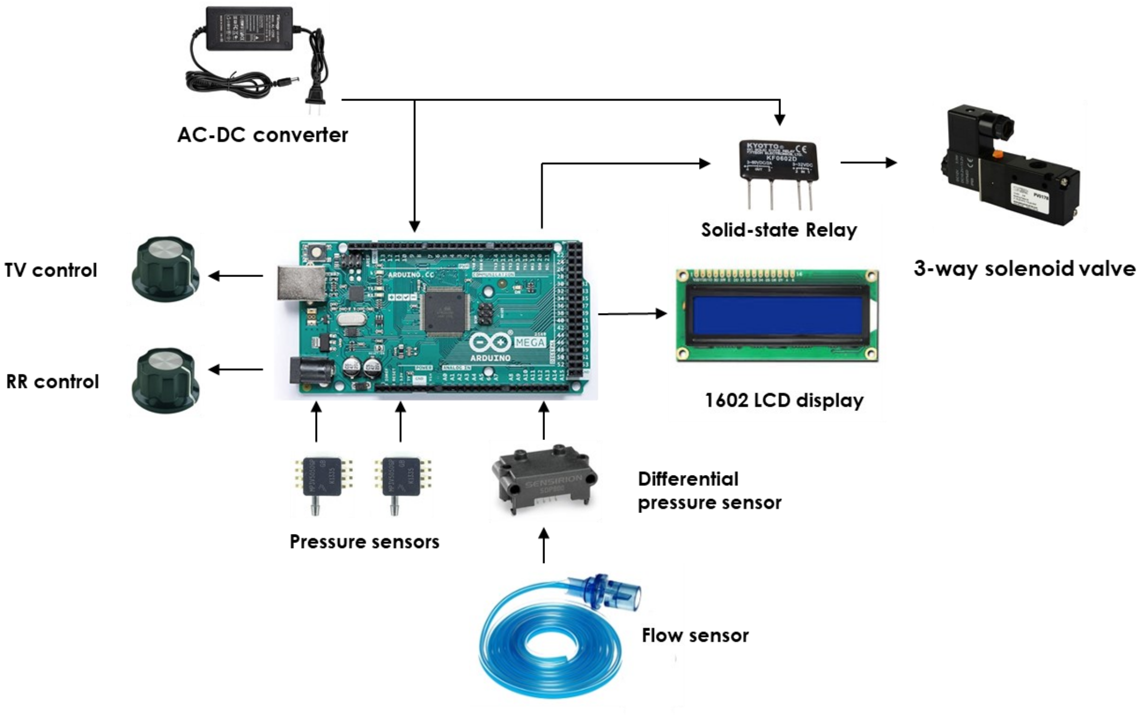

Figure S2. Block diagram of electrical connections.

Components	Unit Cost
AmbuBag with accessories (SunMed AirFlow)	$13.5
PMMA sheet (12" x 12" x 0.5") x 3PCS	$21.5
Solenoid valve (TEMCo PV0178)	$22
Pressure transducer (NXP MPX5050DP) x 2PCS	$20
Differential pressure sensor (Sensirion SDP 816)	$30
Medical flow sensor (Hamilton 281637)	$23
Arduino Mega R3	$33
Electronic components	$30
Extension and other tubing (Home Health Medical Equipment)	$5
AmbuBox chamber accessories	$5
**AmbuBox**	**$266**

Table S1. Summary of the cost breakdown of AmbuBox components.

Tests	Compliance (mL/cmH_2O)	Resistance ($cmH_2O/(L/s)$)	PEEP (cmH_2O)	*RR* (bpm)	t_{infl} (s)
**1**	50	5	5	10 - 20	1.8
**2**	20	5	5	10 - 20	1.8
**3**	20	20	5	10 - 20	1.8
**4**	50	5	5	10	1 – 3
**5**	20	5	5	10	1 – 3
**6**	20	20	5	10	1 – 3
**7**	50	5	5	10 – 30	1 – 3
**8**	20	5	5	10 – 30	1 – 3
**9**	20	20	5	10 – 30	1 – 3
**10**	20	20	10	10	1.8

Table S2. Test conditions of AmbuBox.
